# Noisy galvanic vestibular stimulation modulates spatial memory in young healthy adults

**DOI:** 10.1038/s41598-019-45757-0

**Published:** 2019-06-27

**Authors:** Danica Hilliard, Susanne Passow, Franka Thurm, Nicolas W. Schuck, Alexander Garthe, Gerd Kempermann, Shu-Chen Li

**Affiliations:** 1 German Center for Neurodegenerative Diseases (DZNE), D-01307 Dresden, Germany; 2 CRTD – Center for Regenerative Therapies Dresden, Technische Universität Dresden, D-01307 Dresden, Germany; 3 Faculty of Psychology, Chair of Lifespan Developmental Neuroscience, Technische Universität Dresden, D-01069 Dresden, Germany; 4 Max Planck Research Group NeuroCode, Max Planck Institute for Human Development, D-14195 Berlin, Germany; 5 CeTI – Centre for Tactile Internet with Human-in-the-Loop, Technische Universität Dresden, D-01069 Dresden, Germany

**Keywords:** Cognitive neuroscience, Human behaviour

## Abstract

Hippocampal and striatal circuits play important roles in spatial navigation. These regions integrate environmental information and receive intrinsic afferent inputs from the vestibular system. Past research indicates that galvanic vestibular stimulation (GVS) is a non-invasive technique that modulates hippocampal and striatal activities. There are also evidences for enhanced motor and cognitive functions through GVS. This study extends previous research to investigate whether noisy GVS may improve hippocampal- and striatal-associated aspects of spatial navigation performance. Using a virtual navigation task, we examined effects of noisy GVS on spatial learning and memory. To probe the participants’ sensitivity to hippocampal- or striatal-associated spatial information, we either enlarged the virtual environment’s boundary or replaced an intra-environmental location cue, respectively. Noisy GVS or sham stimulation was applied online during the learning phase in a within-subject crossover design. The results showed that noisy GVS enhanced spatial learning and the sensitivity foremost to hippocampal-dependent spatial information both in males and females. Individual differences in spatial working memory capacity moderated the effects of GVS, with individuals with lower capacity benefitting more from the stimulation. Furthermore, sex-related differences in GVS effects on the two forms of spatial representations may reflect differences between males and females in preferred spatial strategies.

## Introduction

Goal-directed navigation in spatial environments is a common and important activity in human daily living. Accurate spatial navigation requires efficient integration, storage, and evaluation of different types of spatial information that are available in the environment over time. Information provided by external, environmental stimuli (e.g., landmarks, borders/boundaries, or light sources) that are either represented in relation to the body axis (known as the egocentric frame) or in terms of spatial relationships (known as the allocentric frame) is used to guide navigation. In addition, internal sensory information based on self-motion cues (e.g., vestibular information, optic flow, or motor efference) is used to infer relative location or orientation in relation to a reference (known as the idiothetic frame, see Moser *et al*.^1^ for review). Both external and internal inputs need to be integrated and effectively used to support spatial navigation. How such integration occurs is, however, not yet fully clear.

The vestibular system is an evolutionarily old internal sensory system, sometimes subsumed under the heading of ‘proprioception’. Vestibular input and proprioception provide the necessary information about the positions of body parts and the body parts in relation to each other and in space. The vestibular system monitors positions and movements and induces motor reflexes to maintain balance as well as head- and eye alignments, which are also necessary for effective spatial navigation^2^. Deficits of the vestibular system not only cause impaired head-/eye-movements and postural imbalances but also seem to be associated with substantial impairments in hippocampal functions^2–7^. In humans, impairments of the vestibular system have also been found to be associated with hippocampal atrophy^8^. In animal studies, lesioning the vestibular system was associated with dysfunctional hippocampal signaling, such as: attenuated theta-waves^9,10^, eliminated head-direction and place-cell activity^2,6,11,12^ and, therefore, impaired spatial memory.

Non-invasive brain stimulation procedures can be used to externally stimulate the vestibular system. Galvanic vestibular stimulation (GVS) stimulates the vestibular system by weak currents transcutaneously delivered through electrodes that are placed over the mastoid processes behind the ears. Common approaches involve applying either constant currents or randomly fluctuating currents (known as noisy GVS). Electrophysiological studies using neural recordings in animal studies confirm that constant GVS stimulation affect neuronal firing rate in the vestibular nerve (for a review see^13^). Behavioral research on effects of GVS in humans has focused on the role of the vestibular system in sensorimotor integration during postural control^14,15^, gait control and dynamic walking^16^. Other studies have investigated effects of GVS on cognitive functions^5,17^. Of note, available findings suggest that GVS has positive effects on some aspects of cognition, such as face perception and visual facial memory^18,19^. GVS has also been applied to patients with hemispatial neglect^19,20^ and Parkinson’s disease^21,22^. However, depending on examined populations and stimulation protocols (e.g., constant, alternating, or randomly fluctuating currents as well as suprathreshold or subthreshold), the results have been mixed.

Human neuroimaging studies combined with GVS techniques^23–25^ confirmed that vestibular input activates the parietal cortex, the hippocampus, as well as striatal regions, such as the putamen and the caudate (for reviews see^26,27^). Given that (i) GVS activates hippocampal and striatal activities and that (ii) internal vestibular sensory information is also important for spatial learning and memory (besides information from the spatial environment that is processed by the hippocampal formation and extrahippocampal regions, such as the striatum^28–30^), it could be expected that activating the vestibular system by GVS may modulate spatial abilities in humans. To date, however, other than a study using suprathreshold stimulation in humans that yielded an interfering effect on mental rotation ability^31^, no study has investigated whether GVS could improve spatial navigation.

**Figure 1:**
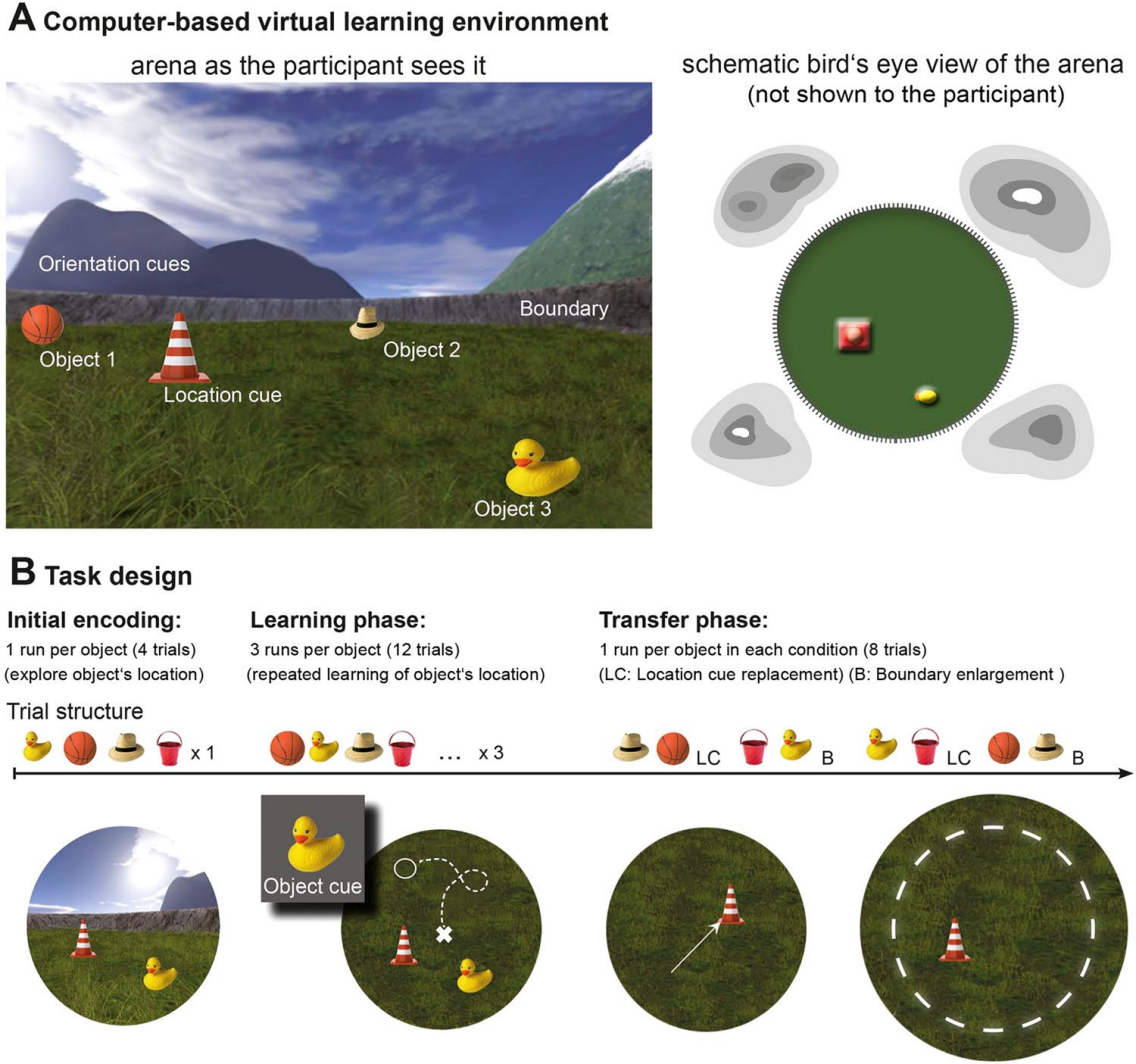
(**A**) Schematic diagram of the task environment is shown from the participant’s (left) and a bird’s eye view (right) perspectives (see text in method section for details about the spatial environment). (**B**) Three phases of the task. In the initial encoding phase, 4 objects were presented one at a time, and participants were instructed to explore and remember their locations. In the learning phase, after probing the search for a given object, participants were instructed to navigate to the memorized object location and drop the object by pressing a button. Afterwards they received feedback about the correct object location. A total of three repetition runs, each consisted of 4 objects probed in a pseudorandomized order were included. The transfer phase comprised the location cue shift (LC) and boundary enlargement (B) conditions that were presented in LC-B-LC-B order. Participants were also instructed to navigate to the memorized location of the cued object, but no feedback was provided anymore.

**Figure 2:**
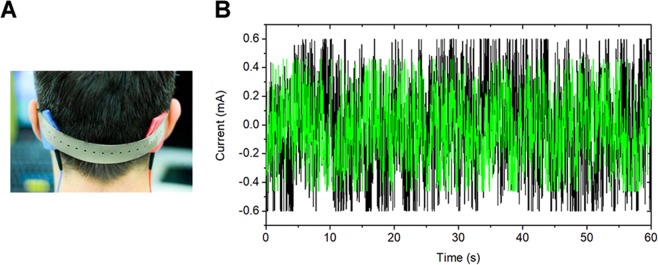
Electrode placements and characteristics of noisy galvanic vestibular stimulation (GVS) signal. (**A**) Placement of stimulating electrodes over the left and right mastoid processes. (**B**) Online low-frequency (0.1–100 Hz) zero-mean random noise stimulation was applied at 80% of the sensory threshold; data shown here reflects a peak-amplitude of 0.6 mA (indicated in black) and 0.43 mA (indicated in green) recorded for a 60 s duration (Voltcraft®, DSO-1062D).

**Figure 3:**
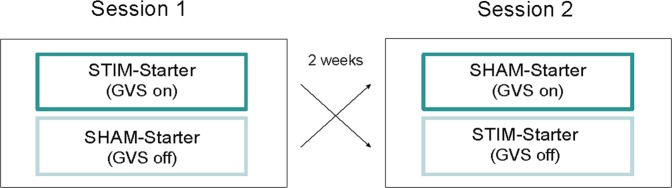
Schematic illustration of the within-subject cross-over design (dark green lines indicate galvanic vestibular stimulation (GVS on), whereas bright green lines indicate sham stimulation (GVS off).

The aim of the current study is thus to investigate the potential performance-enhancing effects of noisy GVS on different aspects of spatial representations by using a computer-based spatial navigation task that allows the assessments of both boundary and location-cue dependent spatial memory (see Fig. 1 and Method section for details). In a within-subject crossover design (see Figs 2 and 3 and Method section for details), we applied noisy GVS or sham stimulation to participants during the spatial navigation task. Noisy GVS follows the principle of stochastic resonance, in which the chance of weak sensory signals (internal vestibular signals in this case) to be detected can be enhanced by adding Gaussian noise^32^. In humans, this type of stimulation protocol has recently been shown to yield positive effects on postural control^33^ and walking^16^. To date, however, potential effects of noisy GVS on spatial navigation abilities have not yet been investigated. As a proof-of-concept study, we explored the effects of GVS on spatial learning and memory in healthy young male and female participants.

## Results

### Comparable group characteristics

Demographic variables, baseline cognitive characteristics as well as GVS-related parameters (i.e., GVS intensity and duration) are shown in Table 1 for STIM- and SHAM-Starters separately for males and females. For males, independent sample *t*-tests revealed no significant differences on any of the cognitive measures between the STIM- and SHAM-Starter groups, except for individual differences in the duration of the GVS stimulation. For females, independent sample *t*-tests also revealed no significant differences on any of the cognitive measures between the STIM- and SHAM-Starter groups, except for the intensity of the GVS stimulation. Crucially, these results confirm that the STIM- and SHAM-starter groups have comparable cognitive abilities.

**Table 1:** Demographic, cognitive, and GVS-related sample characteristics for males and females.

*Control variables*	Males (n = 23)			Females (n = 24)		
	STIM-Starter (n = 11)	SHAM-Starter (n = 12)	*p*-value	STIM-Starter (n = 12)	SHAM-Starter (n = 12)	*p*-value
Age	23.4 (2.6)	23.9 (2.3)	0.59	24.8 (3.6)	24.4 (4.4)	0.80
Years of education	15.6 (2.4)	16.2 (1.6)	0.50	17.9 (3.9)	16.1 (2.7)	0.17
***Cognitive characteristics and GVS-related measures***						
Identical pictures	70.4 (13.7)	72.9 (9.8)	0.59	78.1 (9.4)	70.5 (12.5)	0.11
Identical pictures RT	2131.7 (423.7)	2181.6 (314.2)	0.75	1929.8 (221.8)	2203.7 (437.2)	0.07
Spot a word	65.7 (11.2)	67.4 (14.7)	0.77	70.1 (8.0)	63.2 (11.6)	0.11
Spot a word RT	5738.8 (2057.1)	4970.0 (1636.1)	0.33	4887.0 (1584.7)	3985.3 (940.5)	0.10
Serial Recall	76.3 (21.9)	78.9 (15.6)	0.74	84.9 (9.5)	79.6 (17.6)	0.37
Spatial n-back	82.3 (14.4)	84.6 (6.6)	0.62	65.8 (19.2)	66.7 (24.1)	0.92
Spatial n-back RT	1035.1 (223.9)	953.7 (178.9)	0.34	1202.8 (366.7)	1019.8 (279.5)	0.19
WMT-2	82.3 (13.6)	86.1 (11.7)	0.48	79.2 (15.0)	83.3 (10.3)	0.42
GVS intensity-S1 (in mA)	0.55 (0.25)	0.66 (0.19)	0.26	0.71 (0.23)	0.46 (0.28)	0.03*
GVS intensity-S2 (in mA)	0.68 (0.10)	0.55 (0.21)	0.10	0.59 (0.25)	0.56 (0.26)	0.76
GVS duration (in min)-S1	9.6 (2.9) GVS on	12.9 (6.0) GVS off	0.11	14.7 (6.1) GVS on	14.6 (6.1) GVS off	0.99
GVS duration (in min)-S2	8.8 (3.1) GVS off	13.6 (7.0) GVS on	0.05*	11.8 (4.5) GVS off	11.9 (7.1) GVS on	0.98

Values are presented as mean (standard deviation) with corresponding *p*-values.

Note that the between-group differences in GVS-related parameters are consequences of the self-paced task procedure and individual differences in sensory thresholds. Specifically, the between-group differences in GVS duration during the second session in males indicated that SHAM-starters took slightly longer than the STIM-starters during the encoding and the learning phases, which was simply due to the self-paced nature of the task. Furthermore, since the stimulation intensity of noisy GVS needs to be individually adjusted according to the participant’s sensory threshold^16^, the difference in GVS intensities in females indicated lower threshold levels in female SHAM-Starters than in female STIM-Starters. In control analyses, we checked potential effects of individual differences in these GVS-related parameters by including these parameters as covariates in the statistical models. We also conducted further control analyses that directly compared sample characteristics between males and females to check for potential sex effects in demographic, cognitive and GVS related covariates. Independent sample *t*-tests only revealed significantly better spatial working memory performance (M_males_ = 83.5%, SD_males_ = 10.8; M_females_ = 66.2%, SD_females_ = 21.3, *p* = 0.001) and a slightly shorter GVS duration in session 1 (M_males_ = 11.4 min., SD_males_ = 5.0; M_females_ = 14.7 min., SD_females_ = 6.0, *p* = 0.05) in males than females. Spatial working memory performance correlated negatively with the distance error measure of memorized locations during spatial learning (during SHAM stimulation, *rho* = −0.39, *p < *0.001 in the entire sample; *rho* = −0.27, *p* = 0.02 in males; *rho* = −0.29, *p* = 0.01 in females). Other than these two variables, all other measures did not differ between the two sexes.

Results of all subsequent analyses of variance (ANOVA) were compared with control analyses that included spatial working memory performance, GVS duration and intensity as covariates. The overall patterns of results involving GVS stimulation reported below did not differ between analyses without or with these covariates.

### Significant stimulation effect during the learning phase

**Figure 4:**
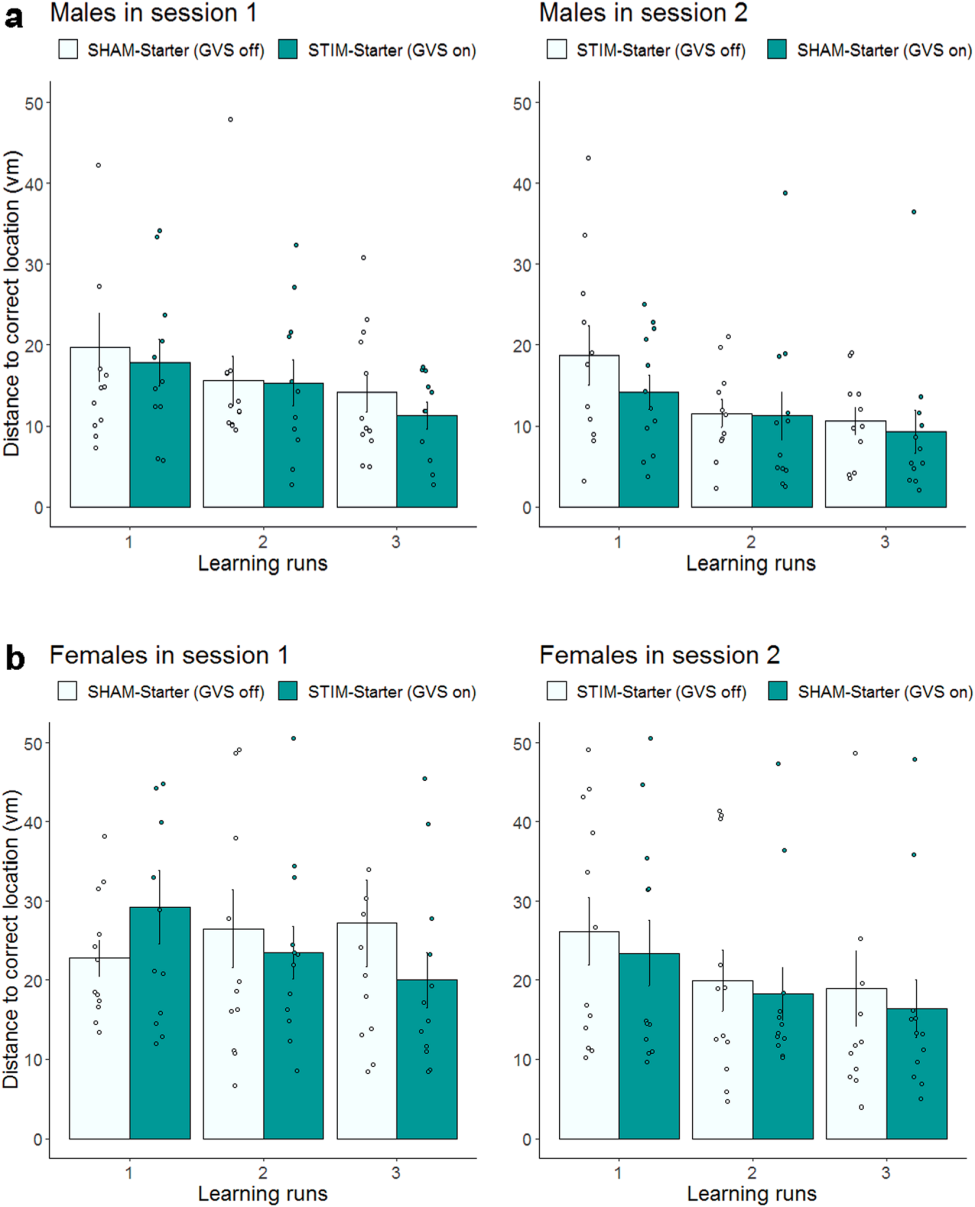
Spatial learning under noisy GVS and sham stimulation in the learning phase for males (**a**) and females (**b**) separately. Distance in vm between memorized location and correct object location for each of the three trials for Session 1 (left panel) and Session 2 (right panel). Dark green indicates GVS was on. Error bars indicate ± 1 SE of the mean. Although spatial learning data were log transformed before analysis, the figures present untransformed values (*N* = 47).

We analyzed the data with a 4-way 2 × 2 × 3 × 2 linear mixed effect model, with stimulation (STIM vs. SHAM), session (S1 vs. S2), run (1–3) as the within-subject factors and sex (female vs. male) as the between-subject factor. The analysis yielded a significant main effect of stimulation, *F*_(1,213)_ = 3.98, *p* = 0.047, ICC = 0.14 (1.8%); session, *F*_(1,213)_ = 20.50, *p* < 0.0001, ICC = 0.30 (8.8%); run, *F*_(2,213)_ = 14.18, *p* < 0.0001, ICC = 0.34 (11.7%); and sex, *F*_(1,45)_ = 14.78, *p* < 0.001, ICC = 0.50 (24,7%). Together, these main effects indicate that vestibular stimulation and learning over time (across runs and sessions) improved spatial memory (see Fig. 4a,b for males and females, respectively). The main effect of sex indicates that males outperformed females in spatial learning, as revealed by their smaller distance error. This sex effect is in line with well-established sex differences in spatial memory (see^34^ for review). All other interaction effects were not significant (all *p* > 0.29).

Comparing results of analyses without and with spatial working memory performance as a covariate showed that, while the main effects of stimulation and run were not affected, the main effect of sex became not significant (*p* = 0.22) after spatial working memory was included in the analysis. The result indicates that part of the sex effect on spatial learning is associated with individual differences in spatial working memory favoring males. Furthermore, in a separate analysis we grouped the participants into high and low spatial working memory group using median split and found a significant 2-way interaction between stimulation and performance group (*p* = 0.03), but a none significant 3-way interaction involving these two factors and sex. This finding indicates that in both sexes individuals with lower spatial working memory capacity benefitted more from GVS stimulation during spatial learning.

### Stimulation effects during the transfer phase

Data from the transfer phase was analyzed first using a 2 × 2 × 2 × 2 linear mixed effect model with four factors: stimulation (STIM vs. SHAM), session (S1 vs. S2), task condition (B vs. LC) as the within-subject factors, and sex (female vs. male) as the between-subject factor. The results revealed a significant main effects of task condition, *F*_(1,127)_ = 6.85, *p* = 0.009, ICC = 0.23 (5.1%) and sex, *F*_(1,45)_ = 5.38, *p* = 0.025, ICC = 0.33 (10.7%). Similar to the data from the learning phase, the sex main effect indicates a performance advantage in males, in line with prior evidence of sex differences in spatial memory^34^. The main effect of task condition revealed performance differences between the boundary and location cue conditions, which is in line with our previous findings^35,36^. Of particular interest here, the analyses also yielded a significant 2-way stimulation × task condition interaction, *F*_(1,127)_ = 3.96, *p* = 0.049, ICC = 0.17 (3%). Furthermore, we also observed a significant 3-way stimulation × session × sex interaction, *F*_(1,127)_ = 6.55, *p* = 0.012, ICC = 0.22 (4.9%), as well as a trend for a stimulation × task condition × sex interaction, *F*_(1,127)_ = 3.07, *p* = 0.082, ICC = 0.15 (2.4%). No further main or interaction effects were observed (all *p*s > 0.13).

**Figure 5:**
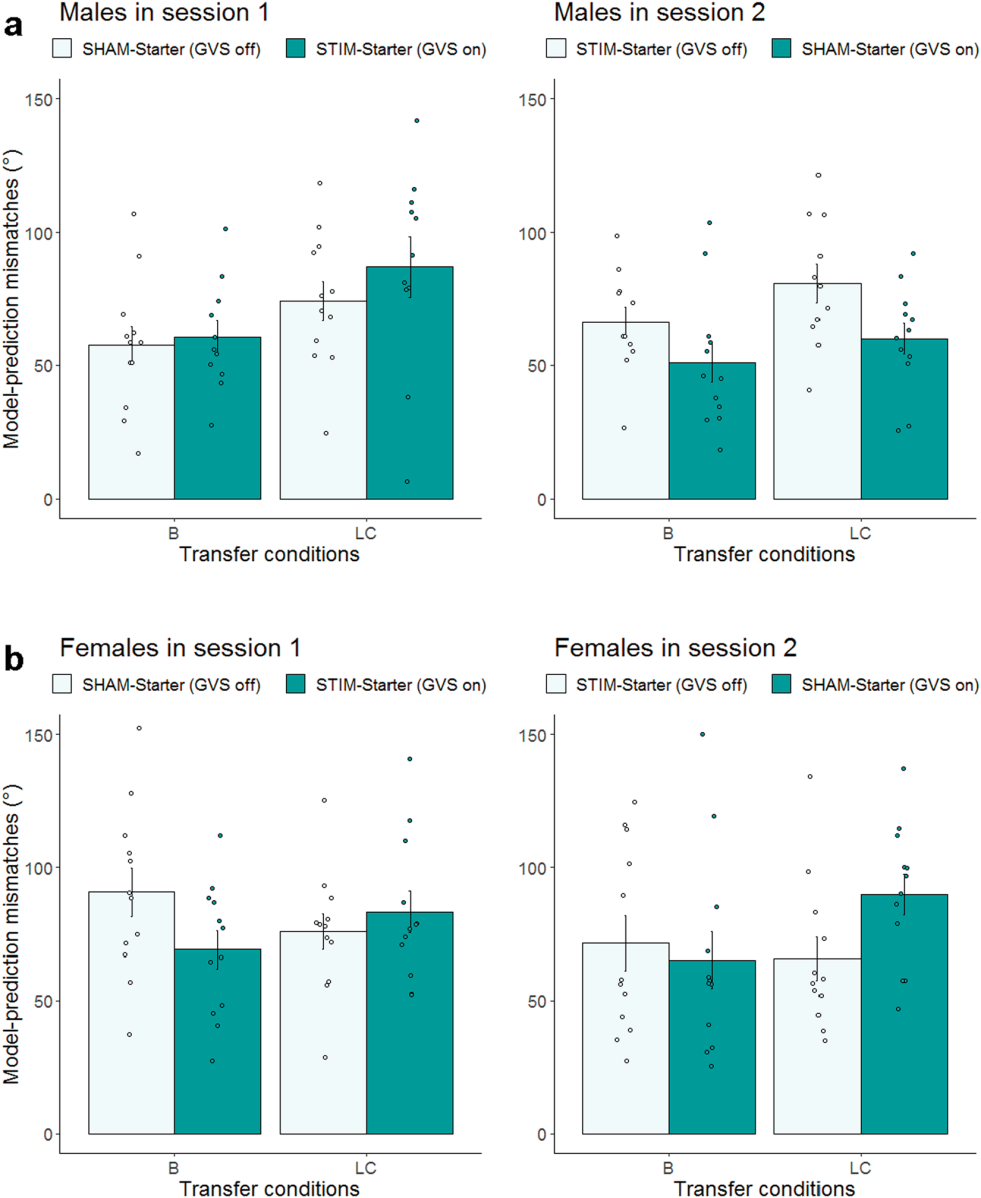
Effects of noisy GVS stimulation on performance during the transfer phase in Session 1 and 2 for males (**a**) and females (**b**) separately. Performance is indicated by the mismatches of behavior vs. model-prediction. Mean differences between observed and model-predicted performance are displayed in angle (°) for the boundary (B) and the location cue (LC) task condition in Session 1 (left panel) and in Session 2 (right panel). Dark green indicates GVS was on. Error bars indicate ± 1 SE of the mean. Although transfer data in females were log transformed before analysis, the (**b**) presents untransformed values (*N* = 24).

Given the main effect of sex and the significant stimulation × session × sex interaction (see Fig. 5a,b for males and females, respectively), follow-up analyses were conducted separately for females and males, with stimulation, session, and task condition as within-subject factors. As shown in Fig. 5a, analyses for the male group revealed a main effect of task condition, *F*_(1,62)_ = 10.86, *p* = 0.002, ICC = 0.39 (14.9%) indicating that during spatial learning male participants were more sensitive to boundary compared to location cue information *t*_(45)_ = −3.20, *p* = 0.002. Although the main effect of stimulation was not significant (*p* = 0.301), we observed a significant stimulation × session interaction, *F*_(1,62)_ = 4.86, *p* = 0.031, ICC = 0.27 (7.3%). This interaction was followed up with post-hoc analyses for each session, which showed that GVS lead to significantly better task performance in session 2, *t*_(43.7)_ = −2.68, *p* = 0.010, whereas in session 1 there was no statistical difference between the male STIM and SHAM group (*p* = 0.37). Since the stimulation × task condition interaction was not significant (*p* = 0.874), the stimulation benefit observed in session 2 was comparable for boundary and location cue information.

In contrast, analyses for the female group yielded a significant stimulation × task condition interaction, *F*_(1,65)_ = 6.088, *p* = 0.016, ICC = 0.29 (8.6%), which indicates that the effect of noisy GVS stimulation differs between the two task conditions. Post-hoc analyses revealed that under sham stimulation, performance did not differ between the two transfer task conditions, suggesting that females were not specifically sensitive to boundary information as males do. Under GVS stimulation, performance differed between the two conditions, indicating that stimulation enhanced females’ sensitivity to boundary-dependent spatial information, *t*_(23)_ = −2.43, *p* = 0.023 [mean mismatch (B_stim_) = 67.16° < mean mismatch (B_sham_) = 81.07°], but lowered their sensitivity to the location cue information, *t*_(23)_ = 2.66, *p* = 0.014 [mean mismatch (LC_stim_) = 86.54° > mean mismatch (LC_sham_) = 70.82°]. All other main and interaction effects were not significant (all *p > *0.14).

Unlike the data from the learning phase, the sex effects observed during the transfer phase remain significant (*p* = 0.03) after controlling for individual differences in spatial working memory performance. Furthermore, the level of spatial working memory performance also did not interact with stimulation (*p* = 0.38) in affecting navigation performance during the transfer phase.

Taken together, results from the learning phase indicate that notwithstanding a sex effect on spatial learning, noisy GVS enhance spatial learning in both sexes during the learning phase. The sex differences in spatial learning are in part associated with females’ lower spatial working memory level. In both sexes, individuals with lower spatial working memory capacity benefitted more from GVS stimulation during spatial learning. Results from the transfer phase show that the effects of GVS on spatial representations associated with boundary or location cue information differed between males and females. In males, although they were more sensitive to boundary than location cue information during SHAM stimulation, GVS-induced improvements in both boundary- and location cue dependent spatial representations, particularly in session 2 (see Fig. 5a, right panel). In females, who were not more sensitive to boundary information during SHAM stimulation, GVS enhanced boundary-, but hindered location cue dependent spatial processing (see Fig. 5b).

## Discussion

Over the past decades various brain stimulation techniques have been developed and applied to support cognitive and sensorimotor functions by modulating cortical excitability. Among these techniques, tDCS (transcranial *direct* current stimulation) and its variants, such as tACS (transcranial *alternating* current stimulation) and GVS (galvanic vestibular stimulation, noisy or DC), are non-invasive and relatively safe methods (for reviews see^17,37^). Differing from tDCS and tACS which are commonly used to stimulate cortical sites, GVS stimulates the vestibular system. Given vestibular inputs to brain regions underlie  spatial cognition as reviewed in the introduction, our aim was therefore to investigate the possibility of modulating spatial navigation performance by means of subsensory noisy GVS. Specifically, in the current proof-of-concept study we had set out to investigate the modulatory effects of noisy GVS on spatial learning and representations that require boundary and location cue based information. In terms of effects of experimental task conditions, in line with previous results^35,36^, mismatches between observed and model-predicted performance were smaller in the boundary than in the location cue-based condition, reflecting that spatial representations in young adults in general rely relatively more on boundary than location cue information, particularly in males. Regarding effects of GVS stimulation, during the learning phase when the participants navigated in the virtual environment to learn and retrieve object locations with feedbacks, the data revealed a main effect of noisy GVS in improving learning performance in both sexes, albeit males’ advantage in spatial learning. Females’ lower spatial learning performance is in part associated with their lower spatial working memory performance. In both sexes, spatial learning of individuals with lower spatial working memory capacity benefitted more by noisy GVS. Since spatial working memory performance correlates positively with spatial learning in our data, this finding could reflect that individuals low in spatial working memory capacity have more room for showing GVS-induced performance improvements. In contrast, regarding effects of GVS stimulation on spatial representations in the conditions of boundary enlargement (B) and location cue shift (LC) in the transfer phase, we observed sex-specific effects. Males benefitted from noisy GVS in both task conditions, in particular after they had obtained prior experiences with the task in an earlier session. After GVS stimulation females showed a behavioral profile that is more sensitive to boundary cues but less to location cue information.

It has been shown in previous fMRI studies that boundary-based spatial learning recruits relatively more hippocampal than extrahippocampal activity, whereas location cue based learning involves striatal activity^35,38^. Our results revealed that vestibular stimulation not only affected performance during the learning phase with feedbacks, noisy GVS also affected spatial representations in the transfer phase when the navigation task became more challenging, with changes in the spatial environment either in the form of boundary enlargement or location cue shift. In males, the beneficial effects observed in the transfer phase were comparable for spatial representations associated with boundary or location cue information, suggesting that noisy GVS influence both hippocampal- and striatal-dependent spatial representations, likely mediated through the vestibular-hippocampal and vestibular-striatal pathways. These effects, however, were moderated by task familiarity and were only observed in session 2. This pattern of result is, in part, in line with our previous finding showing that in patients with Parkinson’s disease dopamine medication (L-DOPA) enhanced hippocampal-associated spatial learning only when the patients had some prior experience with the spatial navigation task in a prior session^36^. In females, noisy GVS enhanced the sensitivity to boundary information, but hampered that to location cue information.

The more general GVS-induced enhancements in striatal- and hippocampal-dependent representations in males, but more specific GVS benefits in only hippocampal-dependent representations in females could, in part, reflect sex differences in preferred navigation strategies. In particular, well-established evidence from previous studies indicates that males and females tend to use different strategies or environmental cues in spatial navigation tasks, which in turn implicate different brain structures^38–40^ (see^41^ for *“Parallel Map Theory”*). Whereas males tend to be more sensitive to geometrical properties of the given environment (hippocampus-related environmental information, such as the *boundary* in our task), females rely more on the location of discrete objects (striatum-related positional cues, such as the *intramaze landmark* in our task). During SHAM stimulation, our findings also show that performance in the boundary condition is better than in the location cue shift condition in males, suggesting that our male participants are more sensitive to hippocampal-dependent information than striatal-dependent location cue information. In contrast, females did not show such a specific sensitivity to hippocampal-dependent boundary information during SHAM stimulation; they, however, also become more sensitive to boundary information after receiving noisy GVS stimulation. The negative effect of GVS stimulation on location cue dependent spatial representation in females may reflect a tradeoff between GVS-induced shifts in spatial processing and females’ reliance on location cue information. As female SHAM-starters became more sensitive to boundary information when they were under GVS stimulation, their sensitivity to location cue based information was attenuated.

Taken together, results of noisy GVS affecting navigation performance that relies either more on boundary or location cue information suggest that vestibular stimulation may modulate hippocampal- and striatal-associated spatial representations. This observation is in line with past brain imaging research showing that vestibular inputs affect hippocampal^25^ and striatal activities^23^. Although studies investigating GVS effects on cognition are accumulating (for reviews see^13,17^), to the best of our knowledge the current findings provide the first empirical evidence of noisy GVS effects in modulating these two facets of human spatial navigation. Our findings at the behavioral level, however, would need to be further investigated in future studies that combine noisy GVS stimulation and brain imaging during spatial navigation, in order to directly examine the effects of GVS on brain activities implicating hippocampal and striatal spatial functions.

Notwithstanding the new insights gained through the results of the current study, as with other non-invasive direct current brain stimulation techniques (e.g., tDCS, tACS),  the outcomes may depend on stimulation protocols and other state-dependent factors, such as the individual’s prior learning, performance level, age, and health status (e.g.^42,43^; for review see^44^). The frequently observed findings of age (e.g.^42^) and performance level (e.g.^43^) moderating tDCS effects on brain functions as well as the sex differences in noisy GVS effects on spatial navigation reported here together point to challenges in generalizing brain stimulation protocols across different populations. Future research will need to systematically compare GVS protocols of different intensity, duration and frequency, in order to titrate stimulation parameters for aging and patient populations.

## Materials and Methods

### Participants

Twenty-three healthy young, right-handed males (aged 20–30 years, mean age 23.7 ± 2.4 years) and 24 females (24.6 ± 3.9 years) gave their informed written consent to participate in the study. Participants were recruited through advertisements on the campus of Technische Universität Dresden. All participants were screened for general health status. None of the participants reported any vestibular, neurologic or other disorders (e.g., balance disorders, psychiatric disorders, metal implants) that would have excluded them from study participation. All participants had normal or corrected-to-normal vision.

To assure that the total sample size has sufficient power to detect noisy GVS effects, we conducted power calculations using G*Power (Version 3.1.9.2) based on effect sizes (partial eta squared i.e., η^2^ partial) estimated from prior studies indicating noisy GVS-related main or interaction effects (i.e.^16,18,33^). The estimated η^2^ partial of these prior studies ranged from 0.15 to 0.36, with a mean of 0.25. We conducted an a-priori power analysis using this effect size f = 0.58 (η^2^ partial = 0.25) for the repeated measures design, with two repeated measurements, a significance level of α = 0.05 (two-tailed), the statistical power (1-β) = 0.95 and an r = 0.25 for the correlation between the repeated measurements. The results of the power analysis suggest that a total sample of 18 participants per group would be required for detecting the main effect of noisy GVS as well as noisy GVS × task condition interaction. Thus, the total sample size of our study (n = 47, 23 males and 24 females) has sufficient power in detecting effects associated with the stimulation. Participants were compensated for study participation (10 €/h). Ethic approval in accordance with the Helsinki declaration for this study was granted by the ethics committee of the TU Dresden (EK 469112015). Informed consents from the participants were obtained prior to study participation. All aspects of the experiment were performed in accordance with the relevant guidelines and regulations.

### Computer-based virtual spatial navigation task

We used a computerized virtual spatial navigation task (cf.^38^) that has been modified and applied to investigate aging-related differences in hippocampal and striatal contributions to spatial memory^35^ or dopaminergic modulation of spatial memory^36^. The task was programmed using UnrealEngine2 Runtime software (Epic Games; http://udn.epicgames.com).

As shown in Fig. 1A, the virtual spatial environment is a circular arena with a grassy field that is surrounded by a visible boundary (a low stone wall) as well as distal orientation cues, such as mountains of different colors, unmoving clouds, and the sun that were projected to infinity behind the stonewall. An intra-environment location cue (i.e., a traffic cone) is presented in a fixed location during the learning phase. The task was presented on the computer screen and participants navigated through the virtual environment by using a joystick. The virtual position (x- and y-coordinates) of the participants were recorded every 100 ms. Distance is expressed in virtual meters (vm), with 1 vm being equal to 62.5 program defined units. The task consisted of three phases: (1) initial encoding during which participants explore the locations of four different everyday objects (e.g., a rubber duck or a ball) in the arena, (2) the learning phase where the participants repeated learns object locations with feedbacks, and (3) the crucial transfer phase, in which either the boundary or the intra-environmental location cue is changed, thus allows the assessments of the hippocampal and striatal-associated spatial memory, respectively (see Fig. 1B). The exploration and learning phases were self-paced.

After familiarizing the participants with joystick-based navigation in a desktop virtual environment, the actual experiment started with one run in the *initial encoding phase*. In each trial, one object was shown and participants had the chance to explore the location of this object while freely moving around in the arena. When the participants felt sufficiently confident about the location of the object, they were asked to virtually “collect” the object by moving the joystick to go over the object (making the object disappear) and then proceed to the next object. The *learning phase* followed the initial object location explorations and consisted of 3 runs, each with 4 trials. In each trial, one out of 4 objects was probed for 4 s on the screen, but disappeared afterwards. After each probe, the participants’ task was to navigate to the memorized object location and press a button to indicate the remembered position for the respective object in the arena. A feedback was then given by showing the correct object location and the participants were given another opportunity to collect the object again. Whereas the objects remain the same across runs, the order with which they were probed within each run was pseudo-randomized. The *transfer phase* started after learning and either the boundary of the circular arena (i.e., the stone wall) or the intra-arena location cue (i.e., position of the traffic cone) was manipulated. In the boundary enlargement (B) condition, the distance (radius) from the center of the arena to the stone wall was expanded by 20% (from 80 vm to 96 vm, thus resulting in an increase of 32 vm in diameter), while the location of the intra-arena location cue was not changed. In the location cue shift (LC) condition, the position of the location cue was shifted away from its original location by about 30 vm, while the boundary remained unchanged. Each of the 4 objects presented in the learning phase was probed in each of the 2 transfer task conditions in a counterbalanced manner for all participants, with 4 trials per each of the two experimental task condition (LC and B). The task was performed in both sessions using two task versions with different objects and object locations.

Three performance measures were derived from the collected data: The raw behavioral data recorded as the Euclidean distance (in vm) between the remembered and the actual object locations during the learning phase (the smaller the distance, the better the location memory). Based on a boundary-vector model^45^ and a location cue model developed in previous work (see^35^ for details), two model-based measures were derived to quantify the extents to which the participants relied on boundary or location cue information during spatial learning: the mismatches (measured as angle deviation) between the participants’ performance and the predicted locations derived from the boundary model reflecting hippocampal-associated learning or the predicted locations derived from the location cue model reflecting striatal-associated learning. A larger mismatch indicates less efficient spatial representation based on either of the two types of information.

### Noisy galvanic vestibular stimulation in a within-subject crossover design

The noisy GVS was delivered by a battery-driven current stimulator (neuroConn®, Ilmenau, Germany) through a pair of conductive-rubber electrodes placed in saline-soaked sponges (5×5 cm) that were attached over the participants’ mastoid processes behind the ears (see Fig. 2A). For participants wearing glasses, glasses with metal frames were replaced by all-plastic glasses during GVS application (MediGlasses for fMRI by Cambridge Research System Ltd., Rochester, Kent, UK) to avoid potential interference. The lenses (±6 diopter in 0.5 diopter increments) were adjusted for each eye until the participant reported satisfactory level of corrected vision. The electrical signals consisted of zero-mean random noise signals over time and were within the frequency range of 0.1–100 Hz (see Fig. 2B). The sensory threshold was individually determined for each participant using a stepwise procedure. Given that the intensity of noisy GVS is commonly adjusted to individual sensory thresholds^16^ we determined the GVS sensory threshold for each participant at the beginning of each experimental session. The starting peak-amplitude of the current was set to 0.25 mA and the highest delivered current was set to 1.25 mA. Noisy currents were delivered for 20 s with increments of 0.025 mA until the participant reported a mild tingling sensation at the electrode sites. This procedure was then repeated twice to confirm the sensory threshold. The stimulation intensity level was set to 80% of the individual sensory threshold to ensure sub-threshold stimulation, which has been previously shown to yield positive effects on walking balance in healthy participants (cf.^16^). In the control (SHAM) condition, the signal intensity was set to 0 mA by turning off the GVS stimulator after the individual sensory threshold was determined. Noisy GVS was applied online until the end of the learning phase of the spatial navigation task (see task description for details). Since the stimulation was applied below the participants’ sensory thresholds, none of the participants noticed any difference between STIM and SHAM conditions and no side effects of GVS stimulation were reported. After completion of the second session, participants were debriefed about the order of the stimulation conditions.

Participants were tested in a 2-session within-subject crossover design with 2 weeks between the sessions (see Fig. 3). The order of the two stimulation conditions (STIM vs. SHAM) was counter-balanced: 23 participants (STIM-Starter, n_males_ = 11) received GVS-stimulation in the first and sham-stimulation in the second session, whereas the remaining 24 participants (SHAM-Starter, n_males_ = 12) received the stimulations in the reversed order.

### Cognitive measures of sample characteristics

Besides the main experimental task, we also assessed basic cognitive covariates to control for potential confounding differences between the STIM- and SHAM-starter groups. Specifically, all participants were assessed with a cognitive test battery, measuring baseline individual characteristics in verbal knowledge and perceptual speed (*Spot-the-word*^46^; *Identical pictures test*^47^), episodic memory (*serial recall task*^48^), abstract reasoning (Wiener Matrizentest 2, *WMT-2*^49^), and spatial working memory (*2-Back with mental rotation*^50^).

### Data analysis

Statistical analyses were performed using SPSS (Version 20.0; IBM Corp., Armonk, NY) and RStudio (Version 1.0.153; http://www.rstudio.com). Baseline sample characteristics between the two starter- (STIM-Starters vs. SHAM-Starters) groups were analyzed using Student’s *t*-test. For analyzing effects of GVS on spatial navigation performance, we applied linear mixed-effect models using maximum-likelihood estimation in RStudio (lme from the nlme package in R^51^) and modeled individual differences by allowing random intercepts for each participants. Effect sizes are given as intraclass correlation coefficients (ICC; cf.^52^). To facilitate the interpretation about the percentage of variance associated with a given effect, squared ICC values are also given in the results section^53^.

We first conducted control analyses that compared sample characteristics between the sex group and between STIM- and SHAM-Starters for each sex separately in demographic and cognitive variables, as well as GVS-related measures (GVS intensity and duration; see results summarized in Table 1) to check for potential between-group confounds before analyzing effects of GVS stimulations on spatial navigation performance. GVS-related effects on performance during the learning and transfer phase were analyzed separately. In a crossover design, a significant stimulation × session interaction would reflect a carry-over of stimulation effects from session 1 to session 2, depending on stimulation status in the first session; in other words, a main effect of the stimulation-starter group (e.g.^36,54^). In case of a significant stimulation × session interaction, analyses were then done separately for the two sessions, by comparing effects between the STIM- and SHAM-starter group. The learning phase was analyzed by conducting a 2 (stimulation condition: STIM vs. SHAM) × 2 (session: S1 vs. S2) × 3 (run: 1–3) × 2 (sex: males vs. females) model. Stimulation, session, and run were within-subject factor, with sex being the between-subject factor. For data from the transfer phase, we conducted a 2 (stimulation: STIM vs. SHAM) × 2 (session: S1 vs. S2) × 2 (experimental task condition: boundary/B vs. location cue/LC) × 2 (sex: males vs. females) model. The normality of the distribution of all models’ residuals was examined using the Shapiro-Wilk test (*W* statistic) as well as visual inspection using Q-Q plots. The data of the whole sample during the learning phase and the data of female sample from the transfer phase were not normally distributed and were therefore log transformed before analysis. The statistical significance level α was set to 0.05 for all analyses.
